# Impact of Chronic Rhinosinusitis with Nasal Polyposis on IL-12 and IL-8

**DOI:** 10.22038/IJORL.2022.53663.2829

**Published:** 2023-01

**Authors:** Janatas Bussador do Amaral, Andrea Goldwasser David, Luciane Mello, Andre Luis Lacerda Bachi, Richard Louis Voegels, Andrew Thamboo, Rogério Pezato

**Affiliations:** 1 *ENT Research Lab. Department of Otorhinolaryngology-Head and Neck Surgery, Federal University of Sao Paulo, Sao Paulo, Brazil.*; 2 *Department of Otorhinolaryngology-Head and Neck Surgery, Hospital Federal da Lagoa, Rio de Janeiro, Brazil.*; 3 *Post-Graduation Program in Health Sciences, Santo Amaro University (UNISA), Sao Paulo, Brazil.*; 4 *Department of Ophthalmology and Otorhinolaryngology, Faculty of Medicine, University of Sao Paulo, Sao Paulo, Brazil.*; 5 *Division of Rhinology, University of British Columbia, Vancouver, Canada.*

**Keywords:** Aspirin-Induced Asthma, Interleukin-8, Interleukin-12, Nasal Polyps, Nose Diseases.

## Abstract

**Introduction:**

The pathophysiology of Chronic Rhinosinusitis is coordinated by distinct inflammatory reactions in different individuals. Inflammatory environments with a predominance of Th2 lymphocytes tend also to be rich in eosinophils. These environments are common during the formation of nasal polyps associated with aspirin intolerance, which is also marked by an increase in inflammatory mediators, especially IL-4, IL-5, and IL-13. Despite the significance of these inflammatory mediators, the relevance of IL-12 subunits' presence within eosinophilic nasal polyps, however, has been less studied. The current study aims to evaluate the presence of IL-12 subunits, IL-12p40 and IL-12p70, in eosinophilic nasal polyps and their correlations with IL-8 presence.

**Materials and Methods:**

We compared the concentrations of IL-8, IL12p40, and IL12p70 among samples of eosinophilic nasal polypoid tissue, eosinophilic nasal polypoid tissue associated with aspirin intolerance, and healthy nasal mucosa, using an indirect immunoassay (ELISA) kit.

**Results:**

When compared to healthy nasal mucosa, there was a lower concentration of IL-8 in Chronic Rhinosinusitis with Nasal Polyp (CRSwNP) tissue. Aspirin Intolerant polypoid tissue also presented a lower concentration of IL-12 subunits compared to healthy nasal mucosa. There was no significant correlation between IL-8 and IL-12 in the eosinophilic polypoid conditions.

**Conclusion:**

In CRSwNP, there is a reduction in IL-8 and IL-12 subunits compared to control, with a lack of correlation between IL-12 and IL-8. The lack of correlation can be justified by a type two inflammatory storm environment.

## Introduction

Chronic Rhinosinusitis (CRS) is a disease that affects 5 to 12% of the general population and has a significant impact on a patient's quality of life. The disease is defined as inflammation of the nasal and paranasal mucosa, accompanied by facial pressure or pain, post nasal drip, rhinorrhea, nasal congestion, and hypo/anosmia, lasting more than 12 weeks (1).

Due to the cell types and cytokines/ interleukins expressed, CRS with Nasal Polyps (CRSwNP) and CRS without Nasal Polyps have different endotypes. Recent studies demonstrated a heterogeneous inflammation pattern in CRS characterized by T-helper 1 (Th1) lymphocytes and neutrophils, as well as inflammatory mediators, such as IL-5 and IL-17 (type 2 and type 3 inflammation patterns) (2,3). 

Neutrophil recruitment is generally determined by signals from tissue damage, epithelial discharge of IL-8, and microbial stimulation. In turn, CRSwNP can also present a mixed lymphocyte profile, composed of Th1 and Th2. This is especially prevalent in eastern populations, compared to the predominance of the type 2 inflammation response found in western populations and severe cases (1,4). 

A chronic inflammatory process potentiated by tissue remodeling can generate abnormal growth of the nasal mucosa, resulting in the formation of nasal polyps (1,5,6). Histopathological studies of nasal polyps demonstrate altered extracellular matrix composition, with decreased collagen, inflammatory cell infiltration, pseudocysts, and edema (1,7–9).

In addition to edema, polypoid tissue usually presents inflammatory cellular infiltration, with a predominance of eosinophils and mast cells (1,5,10). 

On the other hand, CRSwNP is characterized by basement membrane thickening and fibrosis (4). New and preferred therapeutic forms act directly on the mechanical dysfunction associated with NP, aiming to increase the interstitial hydrostatic pressure (11,12). However, the vast majority of clinical treatments for CRSwNP are aimed at mitigating the chronic inflammatory process through anti-inflammatory factors and immunoregulators (1). Hence, in order to optimize the most commonly used treatment plans, predominant inflammatory patterns and related interleukins should be studied and understood. Detecting inflammatory response patterns allows us to guide clinical treatment. An example of this research is the distinction of CRSwNP as a type 2 inflammatory reaction, with elevated levels of IL-4, IL-5, and IL-13 in western populations (13,14).

IL-12 and IL-8 suppress IL-4 and Immunoglobulin E (IgE) production and are the main cytokines related to the immune response associated with a type 1 inflammatory reaction (15). IL-12 comprises IL12p40 and IL12p70 subunits and is secreted mainly by dendritic cells, macrophages, and lymphocytes. This interleukin acts within the innate and acquired immune responses, mainly through the differentiation of Th cells into Th1 and stimulating the production of IFN-y (16–20). The release of IL-12p70 induces a type 1 inflammatory response, and in the absence of p35 expression or its free form, IL-12p40 acts as the antagonist for IL-12p70(16). IL12p40 acts as a competitor to the IL-12 cell receptor, creating a feedback mechanism (18). 

By reason of its different cytokine profiles, unlike upper airway (UA) mucosa, lower airway (LA) mucosa presents no emergence of polyps (21,22). 

During desensitization therapy for aspirin intolerant patients, a decrease in lung cytokines related to a type 2 inflammatory response was noted, as well as an increase in cytokines related to a type 1 inflammatory response (IL-12 and IFN-y) (23). Thus, studies on IL-12, which can alter the type of the inflammatory process, have gained importance in the context of CRSwNP.

Few studies have attempted to assess the relationship between IL-12 and IL-8 in the development of CRSwNP (24,25). The majority of the CRSwNP endotypes are characterized by eosinophilia, with an intense inflammatory process, and a higher recurrence rate. Due to the heterogeneity of the type of inflammation in CRSwNP and the importance of IL-12 and IL-8 on the CRSwNP severity, the assessment of these two interleukins is pivotal. 

In the current study, we aim to access the concentrations of IL-12 (subunits IL-12p40 and IL-12p70) and IL-8 in eosinophilic CRSwNP and then evaluate the correlation between these chemokines.

## Materials and Methods

The current study was carried out at the Department of Otorhinolaryngology of the Federal University of São Paulo, and was approved by the local Ethics Committee (number. 79787817.3.0000.5505).

A group of 39 patients with CRSwNP was recruited, divided into CRSwNP without aspirin intolerance (27) and CRSwNP with aspirin intolerance (12) and compared to a group of participants with no nasal inflammatory alterations (35 controls). The CRSwNP tissue was obtained from nasal polypectomy specimens from patients with CRSwNP, according to the 2020 European position paper on Rhinosinusitis and Nasal Polyps. The diagnosis of aspirin intolerance was based on the patients’ clinical history. The control tissue was obtained from patients with nasal obstruction due to pneumatized middle turbinate (concha bullosa) who had undergone middle turbinoplasty. 

Patients with severe systemic diseases, who used immunosuppressants, with a previous history of rhinosinusitis surgery, or those who did not meet the criterion of a minimum 30-day washout period for systemic or topical corticosteroids or vasoconstrictors prior to baseline were excluded from this study.

*Samples*

Tissue fragments were placed in lysis buffer (PBS + 0.2% Tween-20) containing protease inhibitor (Merck, Darmstadt, Germany) in the proportion of 3 μL buffer/μg tissue. The samples were mechanically homogenized at a speed of 10,000 rpm (TissueRuptor, QIAGEN, USA) and then kept at 4°C before being centrifuged at 10,000g for 10 minutes. The supernatant was collected, aliquoted, and stored at -80°C. The tissue was fixed in 10% acetaldehyde for 24 hours at room temperature. Subsequently, the samples were preserved in 70% ethanol at 4°C, and then embedded in paraffin. Four μm thick sections were obtained with a microtome. The sections were then affixed to Superfrost Plus glass slides (Menzel Glaser, Braunschweig, Germany), and then dried for a few hours at 60^o^C. Deparaffinization was performed by successive baths with decreasing concentrations of alcohol (100, 90, and 70%) for 5 minutes, 2 times. Cell nuclei were stained with alum hematoxylin (Lillie-Mayer solution) for 5 minutes and then washed in running water. The tissues were then stained with an eosin solution for 2 minutes, dehydrated, and diaphanized. Histological examinations were performed using a Nikkon E200 microscope at 400x magnification. The absolute number of eosinophils per high power field (HPF) was counted over an average of 10 fields of view, selected from the most inflamed area of tissue. 

Concentrations of IL-12p40, IL-12p70, and IL-8 protein were quantitated in protein extract with Duo Set® ELISA kits (R&D Systems, Minnesota, USA), following the manufacturer’s guidelines. Positive and negative standards and controls were prepared as described in each kit. The concentrations of cytokines were normalized by the total protein, using Bradford’s method.

*Statistics*

The data obtained were analyzed in PASW 18.0 software (IBM Corporation, Armonk, NY, USA). The Mann–Whitney *U *test was used to test for statistically significant between-group differences. P-values ≤0.05 were considered significant.

## Results

 In the CRSwNP group, 91% of specimens had an eosinophil count above 10 eosinophils per high power field, and 100% had an eosinophil count above 5 eosinophils per high power field.

*IL-12p40 and IL-12p70 levels*

**Fig 1:**
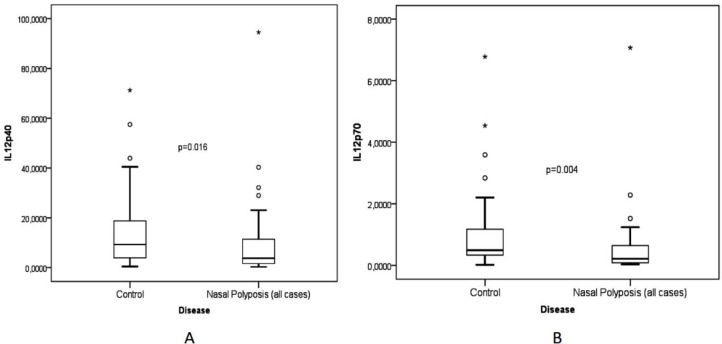
Comparison between IL-12p40 (A) and IL-12p70 (B) in nasal polyposis and control groups. Levels of IL-12p40 and IL-12p70 were significantly lower in the nasal polyposis group than in the control group (p=0.016 and p=0.004, respectively)

**Fig 2:**
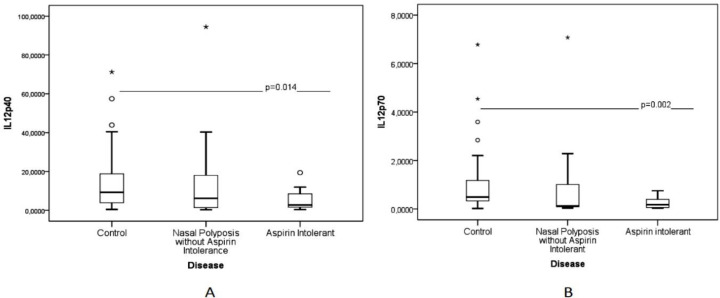
Comparison between IL-12p40 (A) and IL-12p70 (B) in nasal polyposis without aspirin intolerance, nasal polyposis with aspirin intolerance, and control group. Nasal polyposis with aspirin intolerance has lower levels of IL-12p40 and IL-12p70, when compared to healthy nasal mucosa (p=0.014 and p=0.002, respectively)

 Levels of IL-12p40 and IL-12p70 were significantly lower in the CRSwNP group compared to the control group (Figure 1; p=0.016 and p=0.004, respectively). When we divided patients with CRSwNP into groups of CRSwNP with and without aspirin intolerance, we found only the aspirin intolerance subgroup had lower levels of IL-12p40 and IL-12p70 when compared to healthy nasal mucosa (Figure 2; p=0.014 and p=0.002, respectively).

*IL-8 levels*

 Levels of IL-8 were significantly lower in the CRSwNP group than in the control group (p<0.001). When we divided patients with CRSwNP into groups of CRSwNP with and without aspirin intolerance, both groups presented lower levels of IL-8 when compared to the control group (CRSwNP with aspirin intolerance p=0.001 and CRSwNP without aspirin intolerance p<0.001). When we compared the concentrations of IL-8 between CRSwNP with and without aspirin intolerance, we found no significant difference, p=0.589.

*IL-8 levels*

 Levels of IL-8 were significantly lower in the CRSwNP group than in the control group (p<0.001). When we divided patients with CRSwNP into groups of CRSwNP with and without aspirin intolerance, both groups presented lower levels of IL-8 when compared to the control group (CRSwNP with aspirin intolerance p=0.001 and CRSwNP without aspirin intolerance p<0.001). When we compared the concentrations of IL-8 between CRSwNP with and without aspirin intolerance, we found no significant difference, p=0.589.

*Correlations between IL-12p40, IL-12p70 and IL-8 levels*

**Fig 3:**
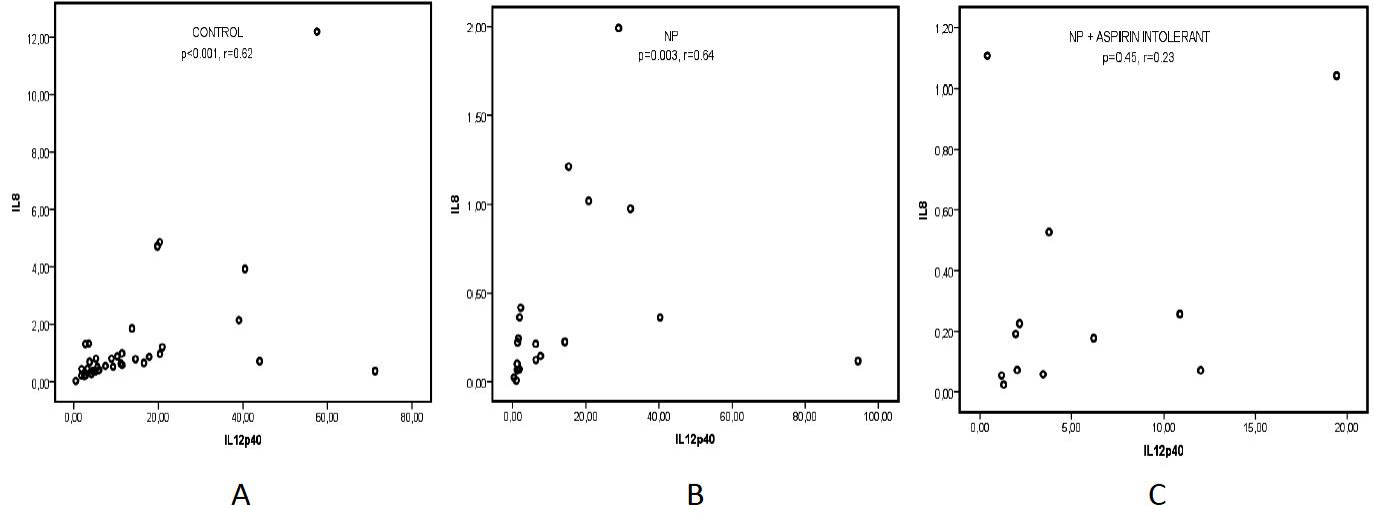
Correlation between IL-12p40 and IL-8 levels in nasal polyposis without aspirin intolerance, nasal polyposis with aspirin intolerance, and control group.** A: **IL-8 has a positive correlation with IL-12p40 in the control group (p<0.001, r=0.62). **B: **IL-8 correlates with IL-12p40 in nasal polyposis without aspirin intolerance (p=0.003, r=0.64). **C:** There is no correlation between IL-12p40 and IL-8 levels in nasal polyposis with aspirin intolerance (p=0.45, r=0,23)

**Fig 4:**
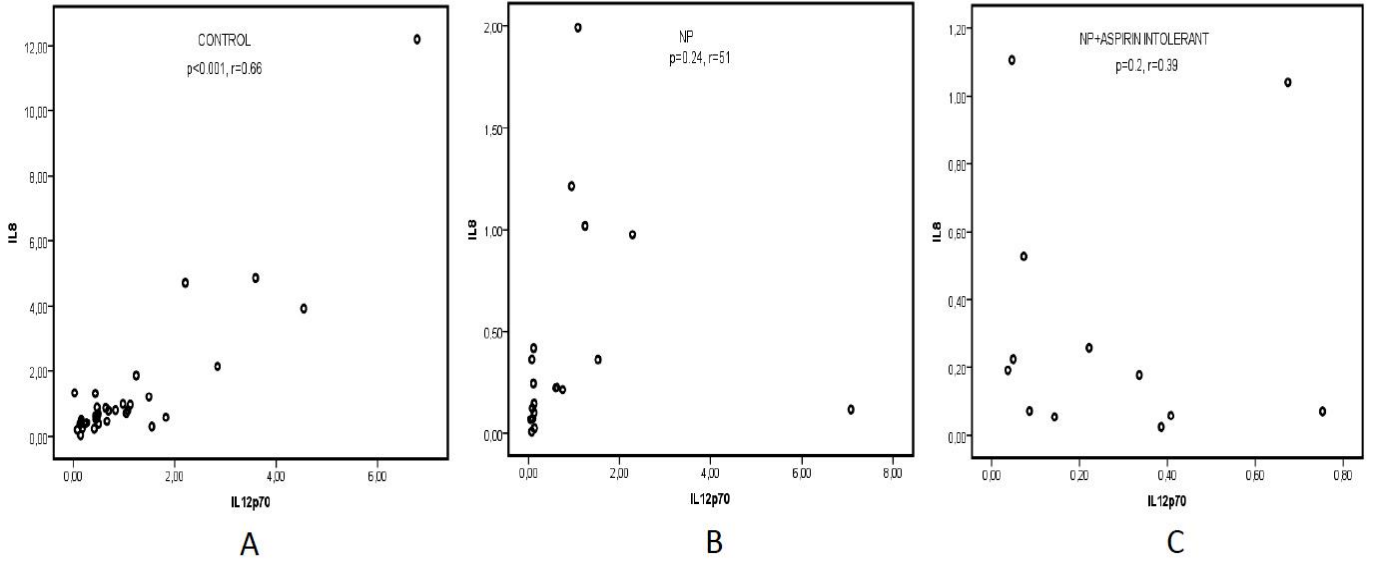
Correlation between IL-12p70 and IL-8 levels in nasal polyposis without aspirin intolerance, nasal polyposis with aspirin intolerance, and control group.** A: **IL-8 has a positive correlation with IL-12p70 in the control group (p<0.001, r=0.66). **B: **There is no correlation between IL-8 and IL-12p70 in nasal polyposis without aspirin intolerance (p<0.24, r=0.51). **C:** There is no correlation between IL-12p70 and IL-8 levels in nasal polyposis with aspirin intolerance (p=0.2, r=0.39)

 IL-8 had a strong, positive correlation with IL-12p40 and IL-12p70 in the control group (p<0.001, r=0.62; p<0.001, r=0.66, respe- ctively) (Figures 3A, 4A). In the subgroup of CRSwNP patients without aspirin intolerance, IL-8 correlated significantly with IL-12p40 but not with IL12p70 (p=0.004, r=0.64; p<0.24, r=0.51, respectively) (Figures 3B, 4B). 

 In the subgroup with aspirin intolerance, IL-8 presented no significant correlation with any IL-12 subunits (Figures 3C, 4C).

## Discussion

Cellular immune response and inflammatory mediators are important protective factors against upper airway infections. When epithelial cells are chronically exposed to pathogens, they secrete cytokines, which are responsible for activating inflammatory pathways, thus initiating the recruitment of immune system cells (12). Interleukin 12 (IL-12) is a pro-inflammatory cytokine secreted by antigen-presenting cells. This cytokine has two heavy chains containing 35-Kda (p35) and 40-KDa (p40), and plays an important role in both adaptive and innate immunity, acting in the differentiation of T helper 1 cells, and regulating immune-mediated cellular responses (17). Cytokines related to a type 2 inflammatory response decrease in aspirin desensitization therapy in patients with aspirin intolerance, while those related to a type 1 response (IL-12 and IFN-γ) tend to increase (23).

In vitro studies have demonstrated the ability of IL-12 to alter the inflammatory ordering of nasal polypoid tissue by increasing IL-21 and CD4 cells that produce IFN-γ through an anti-eosinophilic action (24,25). 

Previous studies reported the impact of intranasal steroids on decreasing inflammatory markers such as IL-4 and IL-5, but not IL-12 (26), demonstrating the need for new drugs able to impact the production of this interleukin.

The present study demonstrated that IL-12 concentration in polypoid tissue in cases of aspirin intolerance is significantly lower when compared to normal nasal mucosa tissue originating in the middle meatus. This finding can be partially explained by our sample of polypoid tissue, which was predominantly eosinophilic and orchestrated by type 2 cells. Although the production of IL-12 was different between polypoid tissue and normal nasal tissue, curiously, there was no change in the IL-12p40 and IL-12p70 ratio when comparing healthy, polypoid, and aspirin intolerance-related mucosa.

IL-8, a proinflammatory cytokine, is closely associated with neutrophil chemoattraction (27,28); surprisingly, we found that IL-8 decreased in our CRSwNP groups. This result is partially explained by our samples being predominantly eosinophilic rather than IL-8 rich neutrophilic samples. Furthermore, the majority of studies that show an increase in IL-8 concentrations and gene expression in CRSwNP used healthy nasal mucosa from the inferior turbinate as control (29,30), increasing the chances of bias (31), whereas we used healthy nasal mucosa from the middle meatus as control. Our study demonstrates that IL-12 and IL-8, two major interleukin mediators of type 1 inflammatory response, may lose their close correlation in environments with a predominance of type 2 immune response. This suggests that a single Th1 interleukin cannot independently initiate an inflammatory response in the hostile environment of CRSwNP where a type 2 inflammatory storm predominates. The findings point to the possibilities of new drugs able to favor IL-12 production and aiming toward a less inflamed environment.

## Conclusion

In the predominantly type 2 inflammatory environment of CRSwNP, there was a decrease in the concentrations of IL-8 and IL-12, as well as its subunits IL-12p70 and IL-12p40, compared to normal nasal tissue. However, differences in IL-12 and its subunits IL-12p70 and IL-12p40 were related only to aspirin intolerance cases. In addition, the loss of the correlation between IL-12 and IL-8 was associated with the CRSwNP severity. 
